# Development of size constancy in children: A review and meta-analysis of size-matching studies

**DOI:** 10.3758/s13423-026-02900-z

**Published:** 2026-04-21

**Authors:** Michael Kavšek

**Affiliations:** Department of Psychology, University of Bonn, Kaiser-Karl-Ring 9, 53111 Bonn, Germany

**Keywords:** Size constancy, Size perception, Size-matching task, Child development

## Abstract

Research on children’s size estimation across varying distances began in the 1920s and has continued to the present. The most prominent method is the size-matching task, in which the participants are asked, for example, to select from several nearby comparison objects the one that corresponds in size to a distant standard object. In this overview, data on the development of size constancy from 24 studies, which provided 245 mean values for size constancy, were analyzed descriptively. Moreover, 102 out of the 245 means were also statistically analyzed. Overall, children of all ages underestimate the size of distant objects. Moreover, size estimations become more accurate with increasing age and with decreasing distance. However, these trends are modulated by several methodological variants of the size-matching paradigm—that is, the impact of the mode of presentation of the comparison stimuli (single/successive versus series presentation of the comparison objects), of the angular separation between standard and comparison objects (simultaneous versus nonsimultaneous visibility of standard and comparison), of the relative position of standard and comparison objects (comparison nearer than standard versus standard nearer than comparison), of the kind of experimental size instructions (objective size versus apparent size instructions), and of viewing conditions (monocular versus binocular viewing conditions). The existing theories on the development of size constancy include the proximal versus constancy mode theory, the metacognitive theory, and the perceptual learning theory. These theories are discussed against the background of the results of the meta-analysis.

## Introduction

**Fig. 1:**
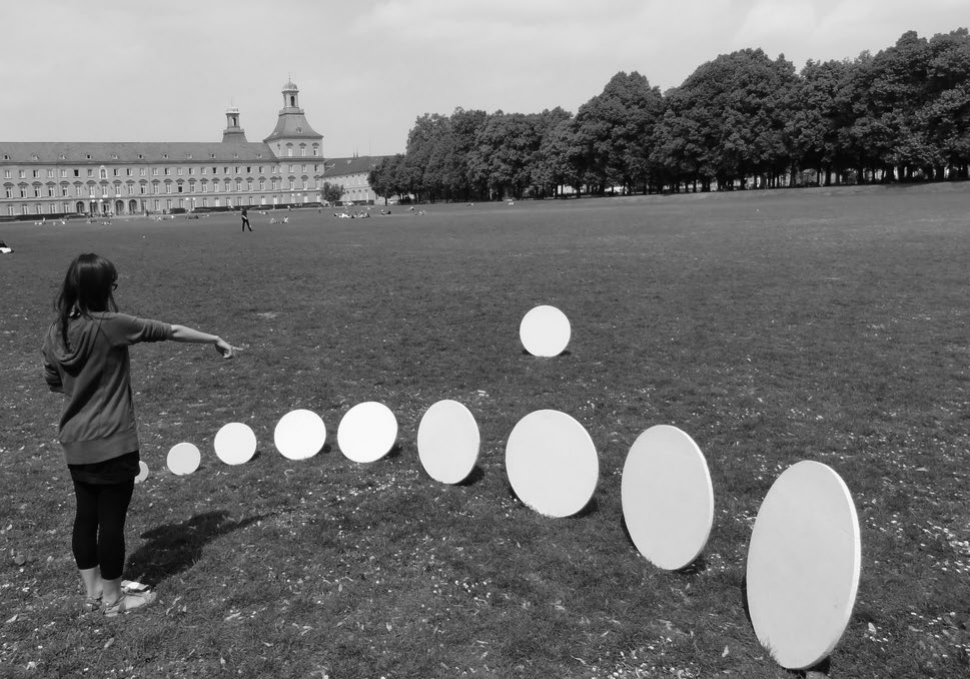
In the size-matching task, the participant selects out of several different-sized comparison objects the one that appears to have the same size as the standard object. In the example, the comparison objects are placed in the foreground and the standard object is more distant

Size constancy is the ability to estimate the correct invariant size of an object over various distances. With increasing distance, the image of an object projected on the observer’s retina decreases. Therefore, in order to perceive the factual, distal size of an object, the observer has to correct the retinal, proximal size of the object by taking into account the presumed distance of the object. The development of size constancy has been experimentally examined as early as 1926 (Beyrl, 1926; Frank, 1926; for a summary of older studies, see Wohlwill, 1960). The basic method for investigating size constancy is the so-called size-matching task. Figure 1 depicts an example of an experimental setup in the size-matching paradigm. In the example, a participant selects from several nearby comparison objects the one that corresponds in size to the distant standard object. The paradigmatic study of Beyrl (1926) provides another specification of the size-matching paradigm. In that study, kind of presentation of the comparison objects and arrangement of comparison and standard differed from the example outlined in Fig. 1. Children ages 2 to 10 years and adults were asked to compare a standard object with each of several comparison objects. However, each of the comparison objects was presented individually together with the standard object. Comparison and standard were arranged such that the standard object was positioned at a distance of 1 m from the participant, while the comparison objects were presented in different experimental sessions at distances of 2, 3, 4, 5, 7 or 11 m. In each stimulus comparison trial, the participant was asked to indicate whether the comparison object was “larger,” “smaller,” or “equal” to the standard object. Each stimulus comparison was repeated 10 times. From the size judgments, Beyrl (1926) determined that perceived object size was generally underestimated with increasing distance. This tendency to conduct underestimation errors or, rather, “underconstancy” was strongest in the youngest participants and decreased with age. For example, in one of the experimental conditions, the adults displayed an underestimation of size of about 1% at the largest distance (11 m). In contrast, in the children younger than 5 years of age, the corresponding underestimation score for 11 m amounted to 40%—that is, they largely underestimated object size. By the age of 9–10 years, underestimation of object size had decreased substantially in the direction of the adult level.

The impact of age and distance on size judgments established by Beyrl (1926) is a basic finding reported in many subsequent studies on size constancy. Younger children tend to underestimate the size of distant (> 10 m) objects. From about 9–10 years of age onward, however, size estimations become more accurate. Adults have been shown to even display a tendency towards “overconstancy”—that is, overestimating the size of distant objects (e.g., Brislin & Leibowitz, 1970; Cohen et al., 1958; Leibowitz et al., 1967; Merriman et al., 2010).

Age and distance are the main variables manipulated in the size-matching studies on size constancy. The youngest children in the Beyrl (1926) study were 2 to 3 years of age. The lowest distance, for which size constancy was examined, amounted to 2 m. In fact, younger children and, of course, infants cannot be tested using the size-matching method due to their still underdeveloped language skills. Moreover, the existing size-matching studies focus on the perception of the size of objects which are located at distances of more than approximately 2 m. Table 1, which provides an overview of the complete data set of the present analysis, shows that the size constancy data on distances < 3 m is underrepresented.

**Table 1:** Mean error scores, broken down by age and by distance

	Complete data set			Data set with *SD*		
Age	*M*	*n*	Distance	*M*	*SD*	*n*
< 5 years	− 11.08	76	< 3 m	–	–	–
	− 19.51	285	3–5 m	− 10.00	3.57	24
	− 40.14	152	6–10 m	–	–	–
	− 52.41	76	> 10 m	–	–	–
5–6 years	− 2.11	49	< 3 m	3.17	2.19	15
	− 9.50	730	3–5 m	− 13,00	13,18	340
	− 15.59	258	6–10 m	− 8.12	8.75	79
	− 23.38	540	> 10 m	− 18.77	15.37	205
7–8 years	− 2.43	20	< 3 m	–	–	–
	− 4.70	511	3–5 m	− 5.23	7.35	319
	− 10.13	323	6–10 m	− 10.60	10.16	131
	− 12.90	259	> 10 m	− 11.68	20.44	179
9–10 years	− 3.87	20	< 3 m	−	−	−
	− 1.80	205	3–5 m	− 0.99	12.75	125
	− 8.10	171	6–10 m	− 9.58	10.18	46
	− 9.06	213	> 10 m	− 7.51	15.88	87
11–13 years	−	−	< 3 m	–	–	–
	− 0.94	325	3–5 m	− 1.00	11.19	305
	− 4.29	212	6–10 m	–	–	–
	− 8.50	96	> 10 m	–	–	–
Adults	0.86	26	< 3 m	1.3	1.56	16
	0.31	674	3–5 m	− 0.01	6.52	387
	− 0.82	313	6–10 m	− 2.63	9.85	65
	0.07	323	> 10 m	2.14	13.93	147

Left half: Mean error scores for the complete data set. Right half: Mean error scores for the data for which standard deviations were unanimously available. The data from those categories that were statistically analyzed are highlighted in gray

The impact of age and distance on size constancy is explained by several theories. Moreover, the research on the development of size constancy is characterized by the application of different experimental concretizations. The goal of the present overview is to describe these theories and experimental variations and to evaluate and discuss them in the light of the existing size-matching studies.

## Theoretical explanations

The most prominent theoretical explanations of the age-related trend from an underestimation of the size of distant objects in children to more accurate size estimations in older children and adults are the perceptual learning theory, the proximal versus constancy mode theory, and the metacognitive theory.

### The proximal versus constancy mode theory

Shallo and Rock (1988) suggest that the underconstancy for large distances found in children is based on a tendency to respond to proximal stimulus information—that is, to retinal size. Retinal size refers to the visual angle subtended by an object. Retinal size varies with an object’s distance. The farther away an object is, the smaller is the image it projects onto the retina. According to the proximal versus constancy mode explanation, adults can voluntarily switch between proximal and constancy judgments. When applying the proximal perception mode, adults base their size judgments on the retinal size of an object. However, adults have learned that the proximal mode does not provide reliable size information. They therefore usually rely on the constancy mode, according to which the factual size of an object is independent of its distance. In contrast, children are in principle also able to employ the constancy mode, but their size estimates are nevertheless strongly biased by the retinal image size. As a consequence, children usually exhibit underconstancy. In their study, Shallo and Rock (1988) presented children ages 5–8 years and young adults a standard object at a distance of 61 m. The participants’ task was to choose that one out of several nearby comparison objects that best matched the standard object in size. In one experimental task, the comparison objects were equidistant from the participant, meaning that they differed in retinal size (i.e., in visual angle). In a second task, the comparison objects were placed at different distances so that they all had the same retinal size (i.e., the same visual angle). When presented with comparison objects that varied in retinal size, the children exhibited underconstancy, while the adults’ size matchings were only slightly underconstant. When presented with comparison objects that were all the same size on the retina, the children’s size estimations were nearly accurate. The adults’ size estimations were slightly overconstant (i.e., they exhibited a slight overestimation or overcompensation of the factual object size). According to the theoretical interpretation of Shallo and Rock (1988), when the comparison objects varied in retinal size, the children’s responses were based primarily and spontaneously on the proximal mode. In contrast, when the comparison objects subtended all the same retinal size, the children were forced to employ the constancy mode. In an attempt to replicate the Shallo and Rock (1988) findings, however, Granrud and Schmechel (2006) determined that young children underestimated the size of the standard objects under both experimental conditions. More specifically, children ages 5 and 6 years displayed underconstancy when the comparison objects differed in retinal size, but also did so when they all had the same retinal size.

According to the proximal versus constancy mode theory, even young children in principle are able to adequately estimate the size of objects at far distances. This ability, however, is suppressed by a spontaneous tendency to rely on retinal-size information. However, this spontaneous tendency can be overcome in favor of the constancy mode by eliminating retinal variance. Shallo and Rock (1988) delineate that the constancy mode refers to an adequate integration of visual angle and information about the object’s distance. More specifically, it consists in the explicit knowledge that a distant object is in fact larger than suggested by its retinal image. In adults, this cognitive factor can even cause overconstancy.

### The metacognitive theory

Shallo and Rock’s (1988) explanatory approach of a cognitively based correction of an object’s retinal size towards its factual, distal size, which can lead to an overestimation of size, is also the core assumption of the metacognitive theory. Basically, the metacognitive theory also assumes that the proximal mode is operative in both children at all ages and in adults. Unlike the proximal versus constancy mode theory, however, young children do not have access to the constancy mode (e.g., Granrud, 2004, 2009; Rapoport, 1967; Wohlwill, 1963). Instead, a cognitively based size compensation is fully applied only from about 9–10 years of age onward. Granrud (2009) tested children ages 5–10 years with nine comparison objects of different sizes that were located at an average distance of 2.74 m. The standard object was placed at a distance of either 6.1 or 61 m. Granrud (2009) established that children with a higher understanding of the influence of distance on the retinal size of an object exhibit a higher size constancy performance at the far distance. When estimating the size of the far distant object, these children typically use the distance compensation strategy and enlarge the retinal size on the basis of the object’s distance. By 9–10 years of age, application of the distance compensation strategy has become the normal case. In contrast, younger children have difficulties to perform a cognitive size compensation and rely more often on retinal size information. Merriman et al. (2010) ascertained that children’s use of the size compensation strategy covariates with their verbal reasoning capabilities. These findings underpin that the development from underconstancy to constancy and overconstancy is not perceptually based but is rather the result of emerging cognitive abilities. Furthermore, the findings indicate that, contrary to the assumptions made by Shallo and Rock (1988), the constancy mode is absent in early childhood.

Overconstancy has been found by several studies with adults (e.g., Carlson, 1960; Epstein, 1963; Gilinsky, 1955). According to the metacognitive theory, overconstancy is due to the use of compensatory strategies. It results from a cognitive compensation that overshoots the adjustment of the diminished proximal, visual size of objects in far distances (e.g., Wohlwill, 1963). Consistent with this assumption, Granrud (2009) observed that some children with a high knowledge about the effect of distance on perceived size exhibited overconstancy as well.

### The perceptual learning theory

The perceptual learning theory (Jenkin & Feallock, 1960; Leibowitz, 1974; Leibowitz et al., 1967) attributes the incorrect judgments of the size of distant objects in children to their underdeveloped size constancy mechanisms. In addition, it is assumed that children are not able to fully exploit the spatial meaning of monocular cues to depth such as texture gradients (Yonas & Hagen, 1973) and linear perspective (Jahoda & McGurk, 1974). Therefore, when binocular depth cues are not available (i.e., under monocular viewing conditions), younger children’s size estimation will be further lowered. As sensitivity to monocular cues increases during childhood, size judgments improve at far distances during childhood. In adults, the size constancy mechanisms are fully developed, as is their sensitivity to monocular depth cues. As a consequence, binocular cues do not substantially contribute to their monocular size matches. To support their theory, Leibowitz et al. (1967) tested children’s and adults’ estimations of the size of objects at different distances under monocular and binocular viewing conditions. Indeed, they established a difference between the two viewing conditions in the accuracy of the size estimation of objects at far distances (15.2 m, 31.5, and 61 m) for children 5 to 11 years of age, but not for adults. More specifically, the children’s size judgments of objects at the far distances were less accurate under monocular than under binocular viewing conditions. In contrast, the adults’ size estimations were equal under monocular and binocular viewing conditions. Moreover, their size estimations were quite overconstant. However, unlike Leibowitz et al. (1967), Kavšek and Granrud (2012) observed no significant effect of monocular versus binocular viewing conditions on younger and older children’s and adults’ size estimations of objects at far (61 m) and near (6.1 m) distances (see also Holway & Boring, 1941).

### The impact of depth cues

The perceptual learning theory points to the role of monocular and binocular distance cues in size constancy. While monocular depth cues are effective at all distances, binocular disparity is most effective at distances up to several meters (Cutting & Vishton, 1995). Nevertheless, it has been shown to also contribute to the perception of depth at very large distances (e.g., Palmisano et al., 2010). At very low distances, up to about 2 m, vergence and accommodation are reliable cues to distance (e.g., Leibowitz & Moore, 1966; Tresilian et al., 1999; Viguier et al., 2001).

## Experimental variations

### Mode of presentation of the comparison objects: Single versus series presentation

A first variation concerns the number of comparison stimuli presented at once. For example, in the Leibowitz et al. (1967) study, the participants were presented with a standard object and only one comparison object at a time. After comparing the sizes of the two objects, the comparison object was replaced by another comparison object and the participant was asked to again make a comparative judgment (see also Beyrl, 1926). In contrast, Kavšek and Granrud (2012) presented the standard object together with all comparison objects, and the participant was asked to select the comparison object that best matched the size of the standard object. In the research literature, the pairwise presentation method or, rather, the consecutive presentation of the comparison objects together with the standard, as carried out by Leibowitz et al. (1967) and Beyrl (1926), is termed constancy, singular, single, or successive method. The alternative presentation method, in which the whole series of comparison objects is presented together with the standard, is called simultaneous or series method (e.g., Burzlaff, 1931; Lambercier, 1946a, 1946b).

### Angular separation between standard and comparison objects: Simultaneous versus nonsimultaneous visibility of the standard and the comparison stimuli

Several studies manipulated the angular separation between comparison and standard. As a result, both the standard and the comparison could be either captured at a glance—that is, they were simultaneously visible, or could only be inspected by looking back and forth (i.e., they could only be visually captured one after the other; e.g., Brislin & Leibowitz, 1970; Jenkin & Feallock, 1960; Piaget & Lambercier, 1943a). With a far distance of 61 m, Brislin and Leibowitz (1970) found generally strong size underestimations in both children 6 to 12.5 years of age and adults. Furthermore, when the standard and comparison objects were simultaneously visible, underconstancy was less pronounced than when the objects could only be captured with separate glances. Brislin and Leibowitz (1970) speculate that the better size constancy scores with a small angular object separation is an effect of experience. In their opinion, situations in which objects are close together are more familiar. Participants are therefore more experienced in the use of size and distances cues in these situations than in situations with objects that are separated by large gaps.

### Relative position of standard and comparison objects: Comparison objects nearer versus farther away than standard objects

A further variation of the experimental stimuli is their relative position to the observer. More specifically, the comparison objects can be either closer to the participant than the standard (e.g., Granrud, 2009; see Fig. 1) or positions of comparison and standard are reversed (e.g., Beyrl, 1926). The distance relevant for determining size constancy is that of the more distant object. If the standard object is more distant than the comparison objects (e.g., Granrud, 2009), selection of a comparison that is physically smaller than the standard indicates underconstancy and selection of a comparison that is physically larger than the standard indicates overconstancy. If, however, the comparison objects are more distant than the standard (e.g., Beyrl, 1926), selection of a comparison that is physically smaller than the nearby standard indicates overconstancy and selection of a comparison that is physically larger than the standard indicates underconstancy. Piaget and Lambercier (1943a) assessed adults and 5- to 8-year-old children with both relative positions of the experimental stimuli. Distances of the objects were limited to the close range below 3.10 m. When the standard object was nearer than the comparison object, size estimations given by the children were underconstant. In the reverse case, when the comparison was closer than the standard, the children’s size estimations were overconstant. The adults displayed overconstancy in both cases with a greater degree of overconstancy, when the comparison was closer to the participant than the standard.

### Kind of experimental size instructions: Objective versus apparent size instructions

Rapoport (1967) tested the effects of both objective and apparent size instructions in participants 5 to 20 years of age. Under objective size instructions, the participants are asked to indicate the real, distal size of an object. Under apparent size instructions, the participants are asked to specify the proximal size (i.e., the size that the object appears to have). Overall, there was a significant age-related trend for increasing size constancy under objective size instructions but not under apparent size instructions. Moreover, Rapoport (1967) observed that children ages 5 to 9 years displayed quite similar object size underestimations under both objective size and apparent size instructions. From 10 years of age onward, however, the participants produced less underconstant size estimates under objective size instructions than under apparent size instructions.

## Sampling

The studies outlined so far point out that there have been proposed competing theories on the development of size constancy. In addition, several methodological variations of the size-matching task have been used in the existing experimental studies, some of which produced contradictory findings. Furthermore, a comparison of studies is often made difficult by the fact that they investigated different object distances. The goal of the present overview was to discuss the existing theories on the development of size constancy and to examine the impact of the stimulus presentation mode (single/successive versus series presentation of the comparison objects), the angular separation between standard and comparison object (simultaneous versus non-simultaneous visibility of standard and comparison), the relative position of standard and comparison objects (comparison nearer than standard versus standard nearer than comparison), the kind of experimental size instructions (objective size versus apparent size instructions), and viewing conditions (monocular versus binocular viewing conditions). These factors will be analyzed within the framework of age differences and distance variations between observer and stimulus target. The impact of the variables listed was scrutinized by comparing the pooled mean scores from the existing studies.

In the first step of the meta-analysis, PsychINFO and the bibliographies of pertinent papers were searched for journal articles, book chapters, and books reporting on experimental studies on size constancy development in children or in children and adults.

In order to conduct descriptive and statistical comparisons of the experimental findings, mean size judgments (*M*), standard deviations (*SD*) associated with these means, and number of size judgments (*n*) for each sample were extracted. To bring the mean size estimations to a common denominator (i.e., to make them comparable), the means were transformed into mean percentage error values, defined as follows:

$$mean\, \%\, error=\left[\left(perceived \,stimulus\, size - actual\, stimulus\, size\right) / actual\, stimulus\, size\right]\times\,100.$$

These mean error scores represent the percentages by which participants under- or overestimated the actual size of a standard stimulus. Negative error values indicate underconstancy, positive error values indicate overconstancy, and a value of 0 indicates accurate size estimation (i.e., perfect size constancy). In a number of studies, means and standard deviations were missing. Whenever possible, these missing parameters were estimated from existing median and range scores and from the number of size judgments (Hozo et al., 2005; see also Furukawa et al., 2006).

Overall, we managed to calculate mean percentage errors for 24 studies with an overall of 6817 size judgments. These size judgments were distributed over a total of 245 mean error scores. Unfortunately, standard deviations could only be determined for the data reported in 13 out of the 24 studies. Thus, standard deviations for 102 out of the 245 mean error scores were obtained. Number of size judgments for these 102 mean error scores was 3,201. Only a limited data set could hence be used for variance analytical comparisons. Therefore, the findings were interpreted mainly descriptively, and these interpretations were supplemented by an additional statistical analysis, whenever the data base for this analysis appeared to be representative. The tables summarizing the data therefore list both mean error scores for the complete data set and statistical information for the smaller data set, for which all parameters (*M*, *SD*, *n*) needed to conduct variance analyses were given. In addition, graphs illustrate the complete data set. The overall data set is summarized in Table 2. The table is available as both an Excel and a SPSS file (OSF: https://osf.io/4r69f/files/sufw4?view_only=16f6ac8055fc4a10b316214e0742adb6).

**Table 2:**
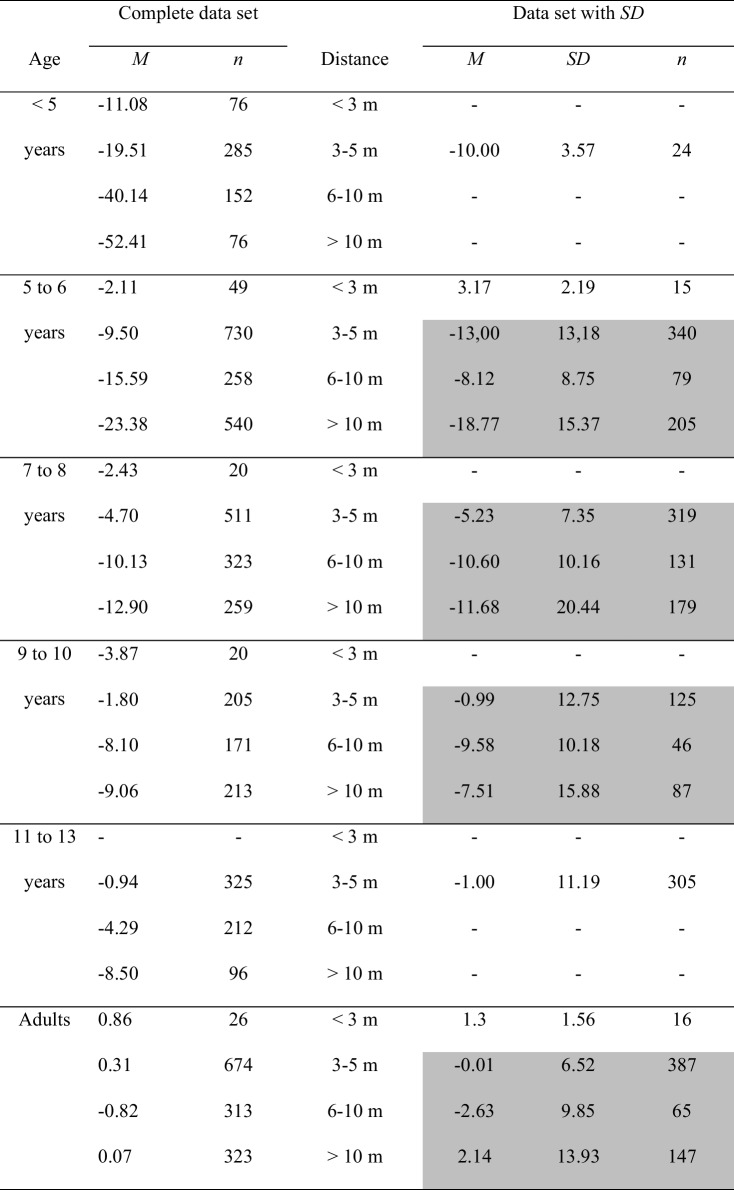
Compilation of the studies summarized in the review. The table contains the mean errors scores, standard deviations, number of size judgments, age groups, and object distances found for each study. Moreover, the table lists the variables manipulated in the studies, that is, the impact of the stimulus presentation mode (single/successive versus series presentation of the comparison objects), of the angular separation between standard and comparison objects (simultaneous versus non-simultaneous visibility of standard and comparison), of the relative position of standard and comparison objects (comparison nearer than standard versus standard nearer than comparison), of the kind of experimental instructions (objective size versus apparent size instructions), and of viewing conditions (monocular versus binocular viewing conditions)

## Results and discussion

### Age and distance

**Fig. 2:**
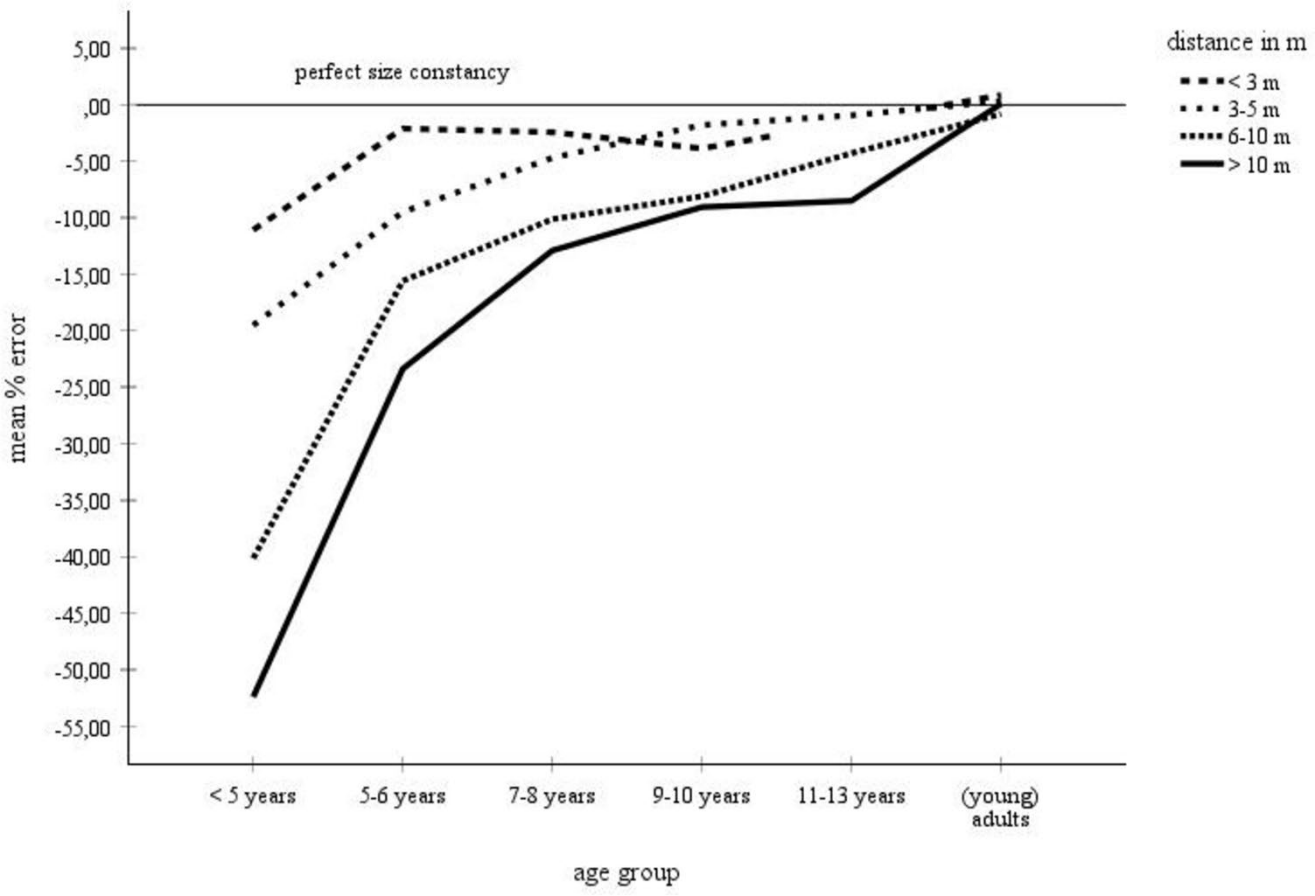
Mean error scores for the complete data set broken down by age and distance. No error bars were entered, because no corresponding information was available for the complete data set

Table 1 and Fig. 2 provide an overview of the collected mean error scores, broken down by age and by distance. Table 1 summarizes the percentage-error means from the complete data set (left side) and for the data set for which standard deviations were available (right side). Age of the participants was classified into six categories (< 5 years, 5–6 years, 7–8 years, 9–10 years, 11–13 years, adults), the object distance variable was subdivided into four categories (< 3 m, 3–5 m, 6–10 m, > 10 m). According to the right part of Table 1, for children ages less than 5 years and children ages 11–13 years, there was only data for the 3–5-m distance category that included standard deviations. Therefore, no statistical analyses could be performed with the data from these age groups. For the remaining age groups, cross-age mean scores and standard deviations were only available for the distance categories 3–5 m and larger. Only this restricted data set—that is, the data set for which mean error scores, number of judgments, and standard deviations, were available, could be statistically analyzed. These data are highlighted in gray in Table 1.

Descriptively, according to the complete data set summarized in Table 1 and in Fig. 2, children display underconstancy at all distances. Overall, size underestimations become smaller, meaning that size estimates become more accurate, with increasing age and with decreasing distance. Especially, children younger than 5 years make inaccurate size estimates with large degrees of underconstancy at all distances. For example, underconstancy at distances > 10 m amounts to 52%. Also, these children show progressively larger underestimation errors with increasing distance. At 5 years of age, there is a developmental leap towards an extreme improvement in size estimations. Moreover, also beyond this age, size estimations continue to improve for distances greater than 3 m. For example, at the farthest distances (> 10 m), children ages 5–6 years underestimate size by 23%, and children ages 9–10 years and 11–13 years underestimate size by about 9% and 8.5%. Finally, adults make approximately accurate size estimates across all distances.

For distances smaller than 3 m, size underestimation in children < 5 years of age amounts to 11%. Beyond that age, mean percent size errors increase and remain at a rather constant level of − 4% to + 0.9%. That is, from 5–6 years of age onward, size estimation errors are quite accurate at the nearest distances (< 3 m), and little to no age-related differences occur in the accuracy of size estimation.

The descriptive analysis was supported by a statistical assessment of the data for which standard deviations were available—that is, of the data marked in gray in Table 1. A 4 × 3 analysis of variance (ANOVA) with age group (5–6-year-olds, 7-year-olds, 9–10-year-olds, adults) and distance (3–5 m, 6–10 m, > 10 m) as main factors on the mean percentage errors revealed significant (α =.05) main effects of age group, *F*(3, 2098) = 80.18, *p* ≤.001, η_P_^2^ =.10, and of distance, *F*(2, 2098) = 21.69, *p* ≤.001, η_P_^2^ =.02. Also, the interaction between both variables was significant, *F*(6, 2098) = 12.71, *p* ≤.001, η_P_^2^ =.04. The significant effect of age group was due to a decreasing overall underconstancy with increasing age, 5–6-year-olds: *M* =  − 14.28, *SD* = 13.49, 7–8-year-olds: *M* =  − 8.18, *SD* = 12.97, 9–10-year-olds: *M* =  − 4.72, *SD* = 13.48, adults: *M* = 0.23, *SD* = 9.21. Tukey post hoc comparisons between the age groups indicated that all scores differed significantly from each other. The main effect of distance was due to a significant decrease of underconstancy from the 3–5-m distance category, *M* =  − 5.31, *SD* = 9.82, to the 6–10-m distance category, *M* =  − 8.23, *SD* = 9.77, to the distance > 10-m distance category, *M* =  − 10.16, *SD* = 16.77, according to Tukey post hoc tests. Finally, the significant interaction between age and distance indicated that younger children increasingly underestimate size with increasing distance. Adults display much better size estimates and their judgments are quite accurate at all distances. Overall, the variance analytical findings confirm the trends contained in the complete data set.

### Mode of presentation of the comparison objects: Single versus series presentation

**Fig. 3:**
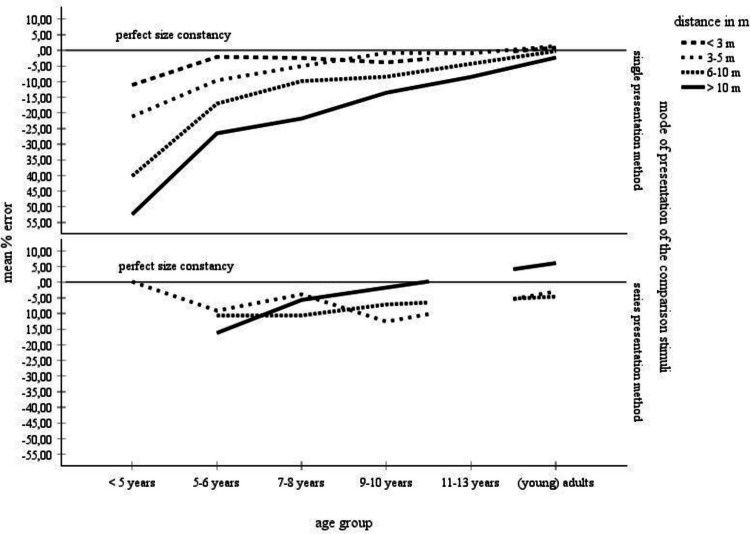
Mean error scores for the complete data set, broken down by method of presentation of the comparison objects and by age and distance. No error bars were entered, because no corresponding information was available for the complete data set

In the single mode of presentation of the comparison objects, the comparisons are presented one after the other and have to be compared individually with the standard. In the series presentation mode, all comparison objects are shown simultaneously together with the standard object and the participant is asked to select the comparison that best matches the standard object. In Table 3, the data collected with the method of single presentation of the comparison objects (comparison objects presented one at a time) are shown in the left panel. The data collected with the method of series presentation of the comparison objects (all comparison objects presented together) are shown in the right panel. The percentage-error means are further subdivided into groups by age and by distance. Moreover, means and sample sizes are shown for both the complete data set and the data set with standard deviations. Again, a part of the data with standard deviations was compared statistically. These data are again shaded in gray. The data from the children ages 9–10 years were not included in the statistical analysis due to large discrepancies between the mean error scores for the complete data set and the mean error scores for the smaller data set—that is, the data set with standard deviations. Due to these discrepancies, the data with standard deviations for the children ages 9–10 years are not representative for the complete data set. For the children ages 5–6 years and 7–8 years and for the adults, the mean error scores of the complete and the smaller data sets were roughly similar. The statistical parameters from these age groups were statistically analyzed for the distances > 3 m. The complete data set is also portrayed in Fig. 3.

**Table 3:**
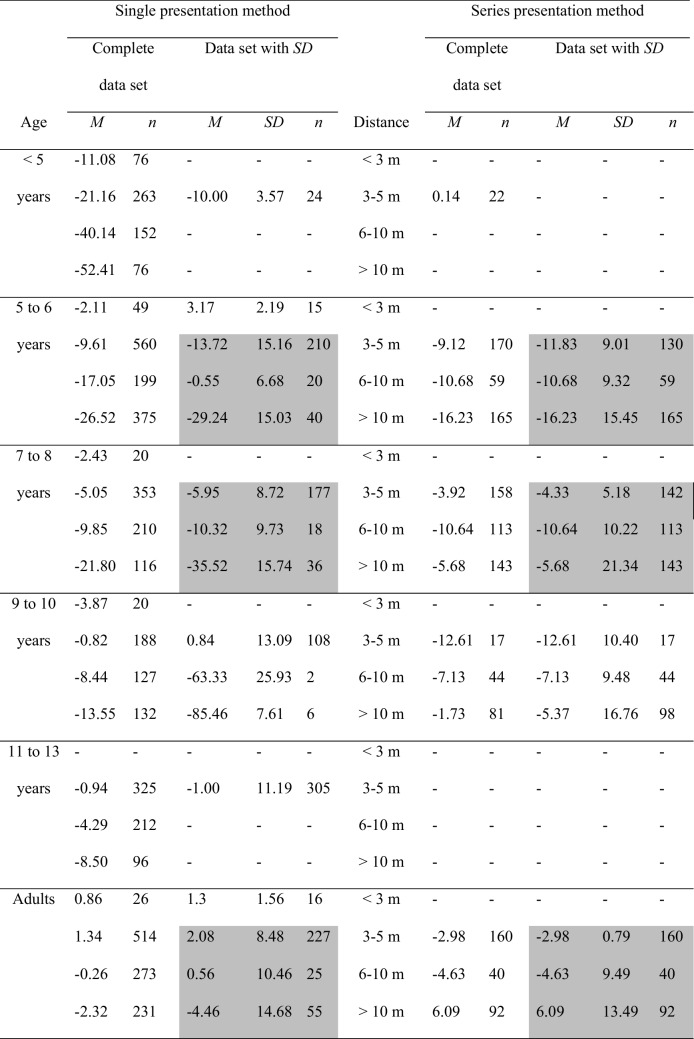
Mean error scores broken down by age and by distance for each mode of presentation of the comparison objects

Left columns: Mean error scores for the complete data set. Right columns: mean error scores for the data with standard deviations. The data for those categories, for which mean error scores and standard deviations were unanimously available, were statistically analyzed. These categories are highlighted in gray

Table 3 and Fig. 3 point out that the developmental trends for the single presentation method largely reflects the overall trends depicted in Fig. 2. First, underconstancy decreases with increasing age. Second, underconstancy increases with increasing distance. As a consequence, the younger the children, the stronger the trend of the distance-related increasing size underestimation. In the adults and in the children older than 9–10 years of age, size judgments are quite accurate for all distances. For the series presentation mode, the developmental curves are much flatter, indicating that both age and distance differences are smaller than in the single presentation mode. Furthermore, in the children, underconstancy is less pronounced in the series than in the single presentation mode. In the adults, size judgments are around the zero value. Unfortunately, there is no data for children younger than 5 years and for children ages 11–13 years, just as there is no data for distances smaller than 3 m. A 2 presentation methods (series versus single presentation method) × 3 age groups (5–6-year-olds, 7–8-year-olds, adults) × 3 distances (3–5 m, 6–10 m, > 10 m) ANOVA compared the percentage-error means. The analysis revealed significant main effects of presentation method, *F*(1, 1884) = 27.71, *p* <.001, η_P_^2^ =.02, of distance, *F*(2, 1884) = 64.76, *p* <.001, η_P_^2^ =.06, and of age, *F*(2, 1884) = 119.95, *p* <.001, η_P_^2^ =.11, as well as significant interactions between age and distance, *F*(4, 1884) = 31.73, *p* <.001, η_P_^2^ =.06, between presentation method and age, *F*(2, 1884) = 17.45, *p* <.001, η_P_^2^ =.02, and between presentation method and distance, *F*(2, 1884) = 93.10, *p* <.001, η_P_^2^ =.09. Moreover, the presentation method by age by distance interaction was significant, *F*(4, 1884) = 7.21, *p* <.001, η_P_^2^ =.02. The significant main effect of the presentation method factor resulted from a slightly overall degree of underconstancy when presenting the comparison objects singly, *M* =  − 7.84, *SD* = 11.81, than when presenting them together, *M* =  − 7.26, *SD* = 12.28, thereby confirming the descriptive data evaluation. The effects of age, distance, and age by distance have been discussed in the age and distance section. The remaining interaction effects found in the analysis were systematically elucidated in additional statistical analyses. Especially, these analyses examined the effects of age and distance for each presentation method separately.

#### Effect of age and distance in the single presentation method

First, the impact of age (5–6-year-olds, 7–8-year-olds, adults) and distance (3–5 m, 6–10 m, > 10 m) were analyzed for the single presentation method (Table 3, left side). According to a 3 × 3 ANOVA, all statistical terms were significant, age: *F*(2, 849) = 72.96, *p* <.001, η_P_^2^ =.15, distance: *F*(2, 849) = 117.67, *p* <.001, η_P_^2^ =.22, and age by distance: *F*(4, 849) = 22.77, *p* <.001, η_P_^2^ =.10. These results reflect the observation that the data for the single presentation mode largely replicate the overall data set described in the section on the effects of age and distance. For the sake of simplicity and clarity, a detailed description of the statistical results was therefore omitted.

#### Effect of age and distance in the series presentation method

A second ANOVA tested the effects of age (5–6-year-olds, 7–8-year-olds, adults) and distance (3–5 m, 6–10 m, > 10 m) on the mean percentage-error scores collected using the series presentation method (Table 3, right side). The effect of age was significant, *F*(2, 1035) = 64.00, *p* <.001, η_P_^2^ =.11, as was the effect of distance, *F*(2, 1035) = 4.59, *p* =.01, η_P_^2^ =.01, and the interaction between age and distance, *F*(4, 1035) = 15.26, *p* <.001, η_P_^2^ =.06.

Tukey post hoc comparisons revealed that size estimation accuracy increases with age, *p* <.001: children of both age groups make more underconstant size estimates than adults, *M* =  − 0.35, *SD* = 8.36. Furthermore, children ages 5–6 years, *M* =  − 13.69, *SD* = 12.52, are less accurate than children ages 7–8 years, *M* =  − 6.61, *SD* = 14.31.

Variation of the mean size estimation errors across the distance categories was generally small. Only the mean errors scores for the 3–5 m category, *M* =  − 6.09, *SD* = 5.76, and the 6–10 m category, *M* =  − 9.52, *SD* = 9.84, differed from each other, *p* <.05, according to Tukey post hoc tests. There was no significant difference between each of these two means and the mean error score for distances over 10 m, *M* =  − 7.33, *SD* = 17.42. Overall, with a series presentation of the comparison objects, participants underestimate size by about 6 to 9%.

The age by distance interaction of the ANOVA was significant. Indeed, the impact of distance was significant in each age group: *F*(2, 351) = 6.58, *p* =.002, η_P_^2^ =.04, for the 5–6 year-olds, *F*(2, 395) = 6.65, *p* =.001, η_P_^2^ =.03, for the 7–8 year-olds, and *F*(2, 289) = 40.51, *p* <.001, η_P_^2^ =.22, for the adults. In the youngest age group, the children ages 5–6 years, Tukey post hoc comparisons indicated that size underestimation was significantly larger in the > 10 m distances than in the lower distances. The difference between the 3–5 m and the 6–10-m distance categories was not significant. In the children ages 7–8 years, the 6–10-m distance category was more underconstant than the 3–5 m and the > 10-m distance categories. In the adults, the judgments for distances > 10 m differed significantly from the judgments for lower distances. Furthermore, unlike the judgements for the lower distances, the judgments for distances beyond 10 m were slightly overconstant.

#### Comparison of the single and the series presentation methods

With both the single and the series presentation methods, size estimation accuracy increases with age. However, several differences between the methods were established. First, application of the single presentation mode results in a significantly overall more pronounced underconstancy than application of the series presentation mode, as indicated descriptively as well as by the significant main effect for method of presentation in the overall ANOVA. Second, size estimates are less accurate at the farthest distances (> 10 m) than at nearer distances (3–5 and 6–10 m) when the single presentation method is used, but not when the series method is used. For the series presentation method, variation of distance exerts only a small effect on the size constancy judgments. Third, different interaction patterns between distance and age were found for the two methods. For the single presentation method, there was a clear distance-related variation of underconstancy during childhood. More specifically, size estimations improved with increasing age and decreasing distance. In particular, the data for the children ages 5–6 years and younger corroborate a strong underconstancy for all distances. In contrast, the impact of distance and of age was smaller in the children who were subjected to the series presentation method. In the adults, single versus series presentation appears to have little effects. Adults’ size estimates are approximately accurate across all distances with both methods.

### Angular separation between standard and comparison objects: Simultaneous versus nonsimultaneous visibility of the standard and the comparison stimuli

**Fig. 4:**
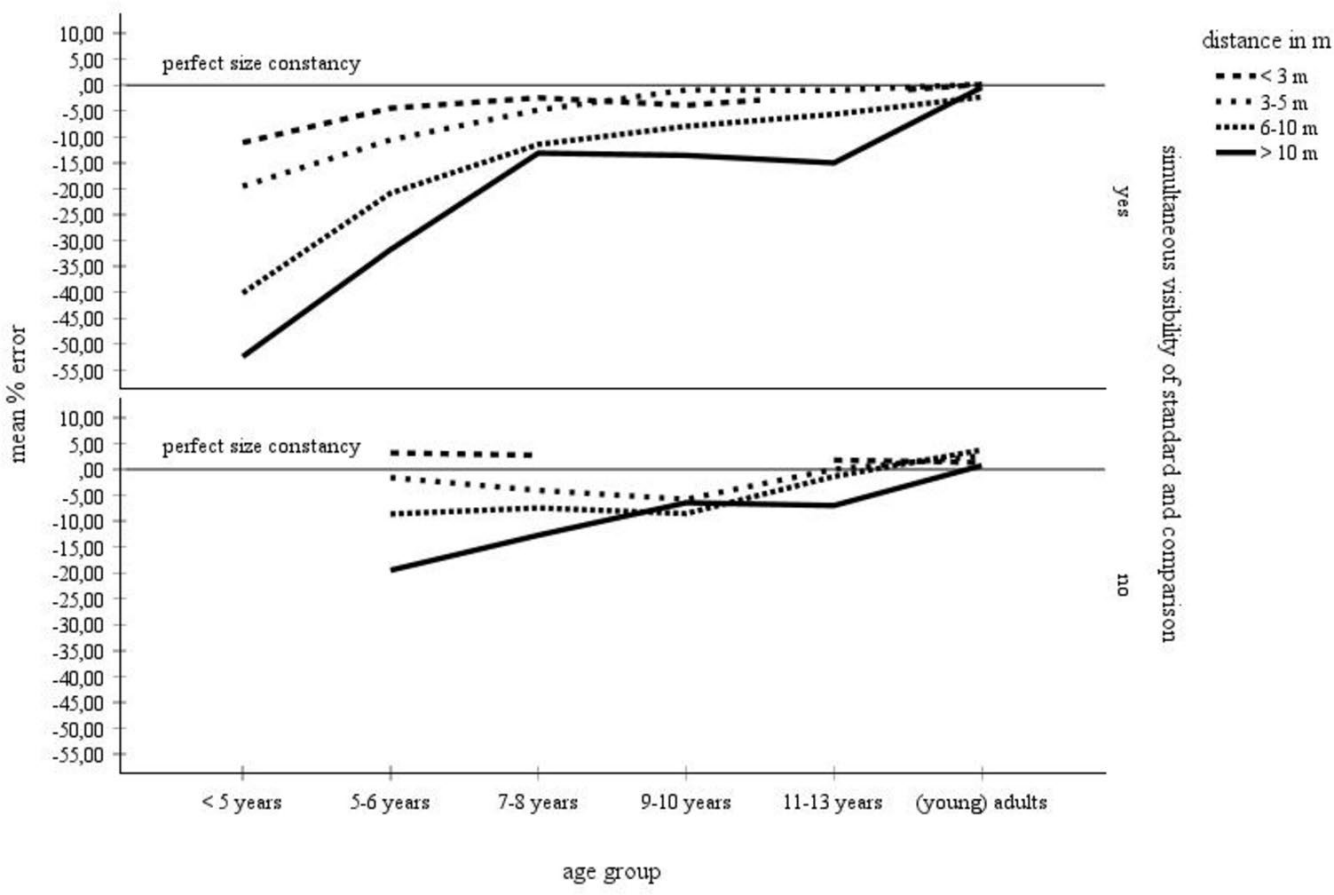
Mean error scores for the complete data set broken by simultaneous visibility of the standard and the comparison stimuli and by age and distance. No error bars were entered, because no corresponding information was available for the complete data set

Table 4 (see also Fig. 4) summarizes the data obtained in studies in which the angular separation between the standard and the comparison stimuli was so small that they all could be seen simultaneously (left part) and in studies in which the separation between the standard and the comparison stimuli was so large that they could not be seen simultaneously (right part). The percentage error means are broken down by the six age groups and the four distance categories. An ANOVA explored the data set with standard deviations. The statistical analysis included the 3–5 m, the 6–10 m, and the > 10-m distance categories for the children 5–10 years of age.

**Table 4:**
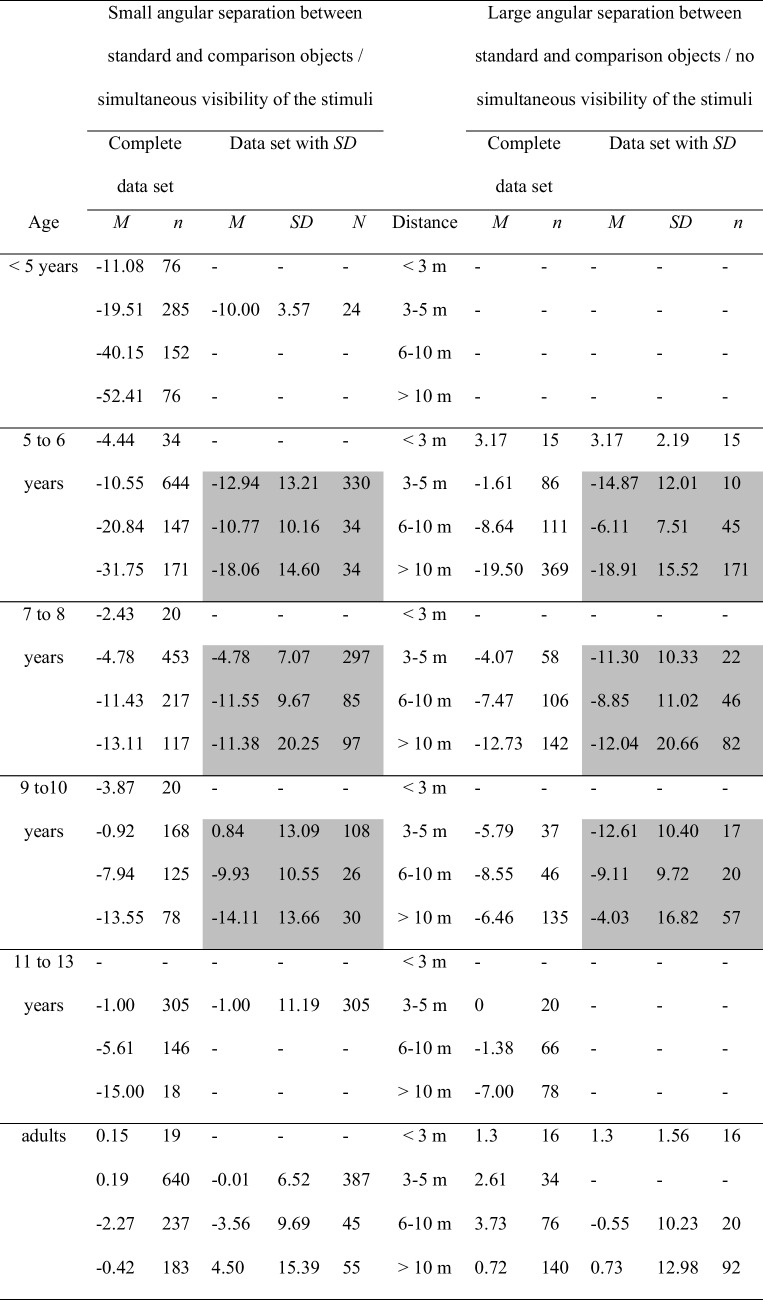
Mean error scores: Simultaneous visibility of the standard and the comparison stimuli by age and by distance

Left columns: Mean error scores for the complete data set. Right columns: mean error scores for the data with standard deviations. The data for those categories, for which mean error scores and standard deviations were unanimously available, were statistically analyzed. These categories are highlighted in gray

The mean percentage errors were compared in a 2 angular separation (small versus large separation between the standard and the comparison stimuli) × 3 age groups (5–6-year-olds, 7–8-year-olds, 9–10-year-olds) × 3 distances (3–5 m, 6–10 m, > 10 m) ANOVA. As expected, the effects of age, *F*(2, 1493) = 8.17, *p* <.001, η_P_^2^ =.01, distance, *F*(2, 1493) = 7.00, *p* =.001, η_P_^2^ =.01, and age by distance, *F*(4, 1493) = 4.06, *p* =.003, η_P_^2^ =.02, were significant. Interestingly, no main effect of angular separation (large versus small) occurred. Moreover, angular separation did not interact significantly with age, but with distance, *F*(2, 1493) = 9.11, *p* <.001, η_P_^2^ =.01, and with both age and distance, *F*(4, 1493) = 3.69, *p* =.005, η_P_^2^ =.01.

The statistical analysis found no evidence that angular separation—that is, the simultaneous visibility of the standard and the comparison stimuli, exerts a significant main effect on the size estimates. However, angular separation interacted with age and distance. Separate analyses show that for small angular stimulus separations, the effects of age, *F*(2, 1032) = 9.94, *p* >.001, η_P_^2^ =.02, distance, *F*(2, 1032) = 30.51, *p* <.001, η_P_^2^ =.06, and age by distance, *F*(4, 1032) = 6.09, *p* >.001, η_P_^2^ =.02, were significant. In contrast, for large angular stimulus separations, only the age by distance interaction reached significance, *F*(4, 461) = 4.62, *p* =.001, η_P_^2^ =.04. These results are largely consistent with the descriptive examination of the complete data set. The complete data set stresses that a simultaneous visibility of the stimuli entails the main effects of age and distance, and the age by distance interaction described in the section on age and distance. In case of large separations between standard and comparison objects, however, distance differences are less pronounced in the children ages 9–10 and 11–13 years. Unfortunately, there was no data for the youngest age group (< 5 years of age). It therefore remains unclear, whether or not the large underconstancy sores observed in children younger than 5 years for the simultaneous stimulus visibility mode also applies in the case of a large separation between standard and comparison. To summarize, the descriptive assessment of Table 4 and Fig. 4 points out that a simultaneous visibility of comparison and standard fosters clear age and distance differences comparable to those outlined in the analysis of the overall data set in Table 1 and Fig. 2. Moreover, these differences flatten out under large stimulus separation conditions—that is, when it is not possible to capture comparison and standard with a single glance.

### Relative position of standard and comparison objects: Comparison objects nearer versus farther away than standard objects

**Fig. 5:**
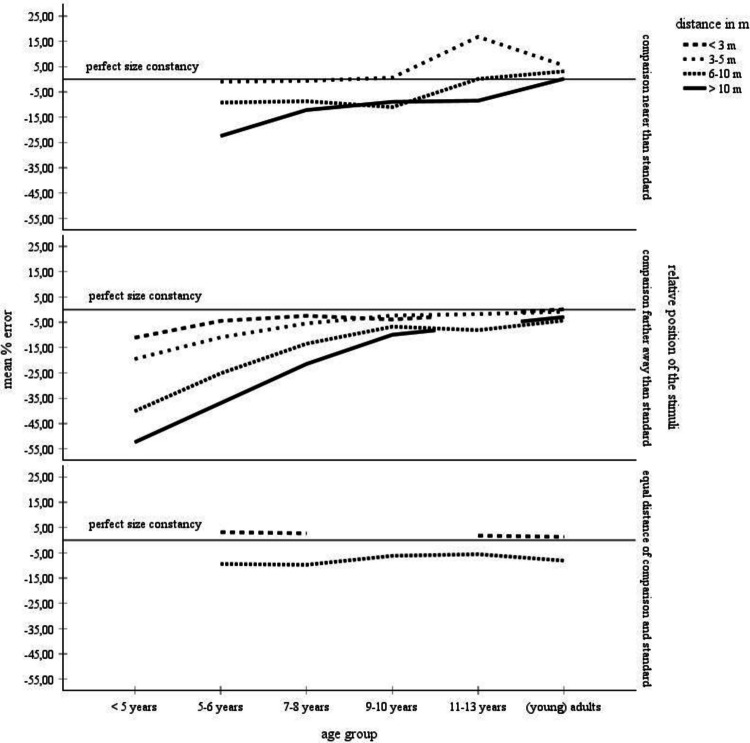
Mean error scores for the complete data set broken by relative position of standard and comparison stimuli and by age and distance. No error bars were entered, because no corresponding information was available for the complete data set

The comparison stimuli can either be nearer or farther away from the observer than the standard objects. Table 5 and Fig. 5 show the combination of this variable with the participants’ age and with stimulus distance. According to the complete data set, at distances > 3 m, children display underconstancy with both relative positions. Size estimates become more accurate until 9–10 years of age when the comparison stimuli are farther away than the standard. In case the comparison stimuli are nearer than the standard, this trend is considerably weaker. Here, the size estimates are less underconstant in general. Moreover, when the comparison objects are farther away than the standard, children’s size estimates become less accurate with increasing distance. Again, this trend is weaker when the comparison stimuli are nearer than the standard. Overall, children display a larger underconstancy when the comparison objects are farther away than the standard objects. Adults tend to very slightly underestimate size when the comparison objects are farther away and to very slightly overestimate size when they are nearer than the standard object. Altogether, the developmental trend that arises when the comparison objects are farther away than the standard is similar to the overall age by distance trend depicted in Table 1 and in Fig. 2. This trend is less pronounced for the reverse situation in which the comparison objects are nearby the observer. The lower part of Fig. 5 also shows data from the studies conducted by Leibowitz et al., (1967; Leibowitz et al., 1972a) and by Piaget and Lambercier (1943b), who presented the standard in the same distance as the comparison. Piaget and Lambercier (1943b) presented the objects in a very near distance of 1–1.8 m and found an overconstancy of 3.17% in children ages 5 years and of 1.3% in adults. Leibowitz et al. (1967), for an object distance of 7.62 m, established an underconstancy of about 11% for children ages 5.5–11.5 years and for adults. Similarly, for the distance of 6.71 m, Leibowitz et al., (1972a) observed mean error scores of − 5.64%, − 8.66%, and − 10.63% in children 5 and 9 years of age and in adults, respectively. That is, even in the case of equidistancy of the experimental stimuli, there is no perfect size constancy. Instead, according to Fig. 5, there is a very slight overconstancy for a very close distance, and underconstancy for a somewhat larger distance.

**Table 5:**
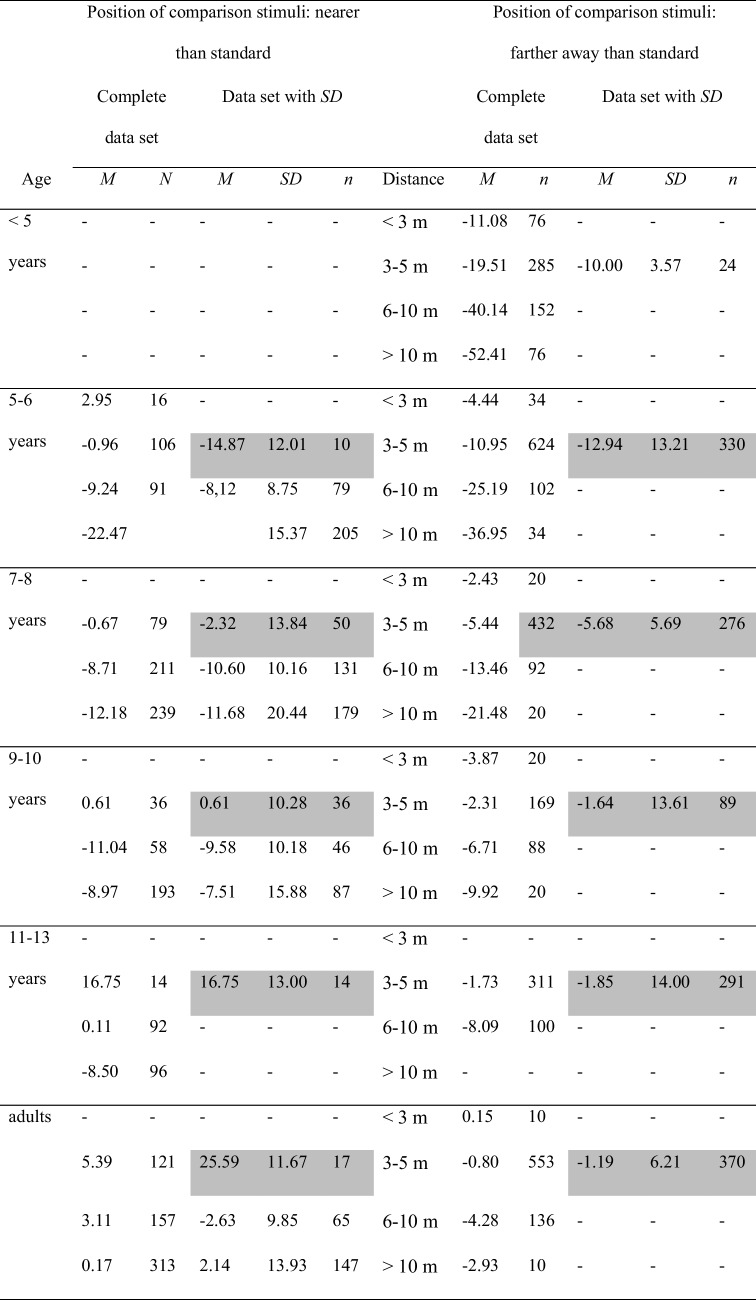
Mean error scores: Relative position of standard and the comparison stimuli by age and by distance

Left column: Mean error scores for the complete data set. Right column: mean error scores for the data with standard deviations. The data for those categories, for which mean error scores and standard deviations were unanimously available, are highlighted in gray

Table 5, right side, shows that there is only a small number of overlapping data in the data set with standard deviations. Therefore, no statistical data analysis was carried out.

### Kind of experimental size instructions: Objective versus apparent size instructions

**Fig. 6:**
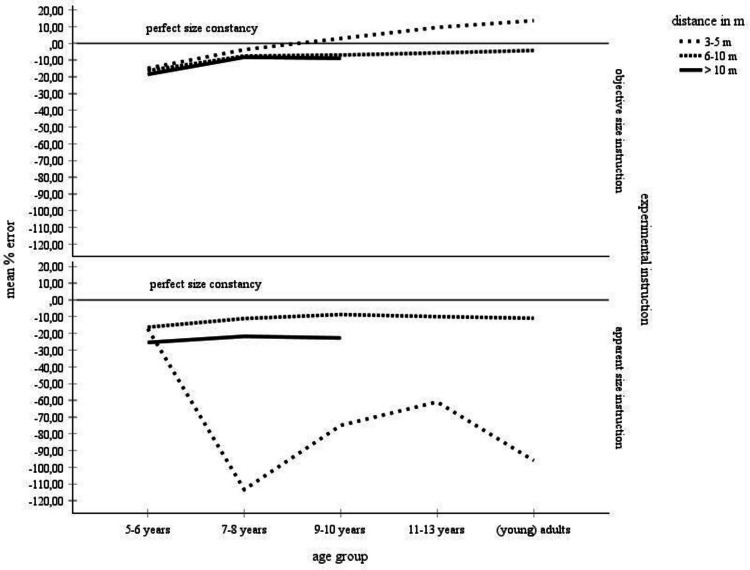
Mean error scores for the complete data set broken by objective size versus apparent size-instructions and by age and distance. No error bars were entered, because no corresponding information was available for the complete data set

Four studies explicitly required participants to make objective and apparent size judgments (Granrud, 2009; Piaget & Lambercier, 1951, 1956; Rapoport, 1967). Granrud (2009) tested the effect of the variation of experimental instructions in children. Rapoport (1967) and both studies conducted by Piaget and Lambercier (1951, 1956) additionally investigated adults. Their findings are summarized in Table 6 and in Fig. 6. Table 6 and Fig. 6 show the distribution of the percentage-error means across experimental size instructions, age, and distance. The data collected using objective size instructions are shown in the left half, the data collected using apparent size instructions are shown in the right half of Table 6. Neither of the studies reported data for children younger than 5 years of age and for distances less than 3 m.

**Table 6:**
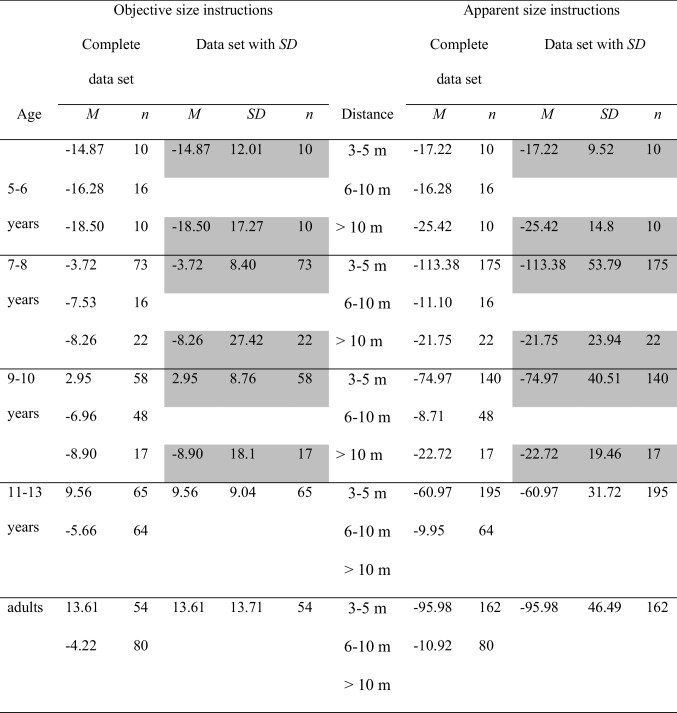
Mean error scores: Objective versus apparent size instructions by age and by distance

Left column: Mean error scores for the complete data set. Right column: mean error scores for the data with standard deviations. The data for those categories, for which mean error scores and standard deviations were unanimously available, were statistically analyzed. These categories are highlighted in gray

For distances 6–10 m and > 10 m, according to Table 6 and Fig. 6, underconstancy is only slightly more pronounced under apparent size instructions than under objective size instructions. In contrast, for the distances 3–5 m, the effect of instructions on the size judgments is very large. Here, apparent size instructions result in a very pronounced underconstancy in the participants ages 7–8 years and older. This effect is due to the data contributed by the studies of Piaget and Lambercier (1951, 1956) who report very negative mean error scores for the 3–5-m distance category in the children 7–14 years of age and in adults. Without their data, the effect of instructions on the size judgments is quite small.

Data that could be statistically analyzed were available for children ages 5–6 years, 7–8 years, and 9–10 years in combination with the 3–5-m and the > 10-m distance categories. The means were compared in a 2 size instructions (objective versus apparent size instructions) × 3 age groups (5–6-year-olds, 7–8-year-olds, 9–10-year-olds) × 2 distances (3–5 m, > 10 m) ANOVA. This analysis found significant main effects of age, *F*(2, 552) = 4.23, *p* =.015, η_P_^2^ =.02, of distance, *F*(1, 552) = 15.89, *p* <.001, η_P_^2^ =.03, and of the size instructions term, *F*(1, 552) = 51.23, *p* <.001, η_P_^2^ =.09. Moreover, all interaction terms were significant. Especially, the size instructions by age by distance interaction reached significance, *F*(2, 552) = 7.38, *p* =.001, η_P_^2^ =.03.

The information gained by the statistical assessment of the smaller data set is fully consistent with the descriptive analysis of the complete data set. The main effect of size instructions indicates that children make significantly more underconstant size estimates with apparent size instructions, *M* =  − 84.57, *SD* = 45.13, than with objective size instructions, *M* =  − 4.04, *SD* = 13.78. The significant interaction between size instructions, age and distance was further investigated by separate statistical analyses for each size instruction. For objective size instructions, both age, *F*(2, 184) = 7.14, *p* =.001, η_P_^2^ =.07, and distance, *F*(1, 184) = 6.22, *p* =.014, η_P_^2^ =.03, reached significance. These effects replicated the general dependency of the mean error scores on age and distance. The interaction between both terms was not significant. For apparent size instructions, all terms were significant: age, *F*(2, 368) = 9.12, *p* <.001, η_P_^2^ =.05, distance: *F*(1, 368) = 28.39, *p* <.001, η_P_^2^ =.07, and age by distance: *F*(2, 368) = 10.53, *p* <.001, η_P_^2^ =.05. The interaction between age and distance reflects the strong underconstancy in the children 7–8 and 9–10, but not in the children 5–6 years of age in the 3–5-m distance category (see also Fig. 6).

### Viewing conditions

Only two studies included both monocular and binocular viewing conditions (Kavšek & Granrud, 2012; Leibowitz et al., 1967). Only the study conducted by Kavšek and Granrud (2012) reported standard deviations. Therefore, the data from the two studies could not be subjected to a joint statistical data analysis.

**Fig. 7:**
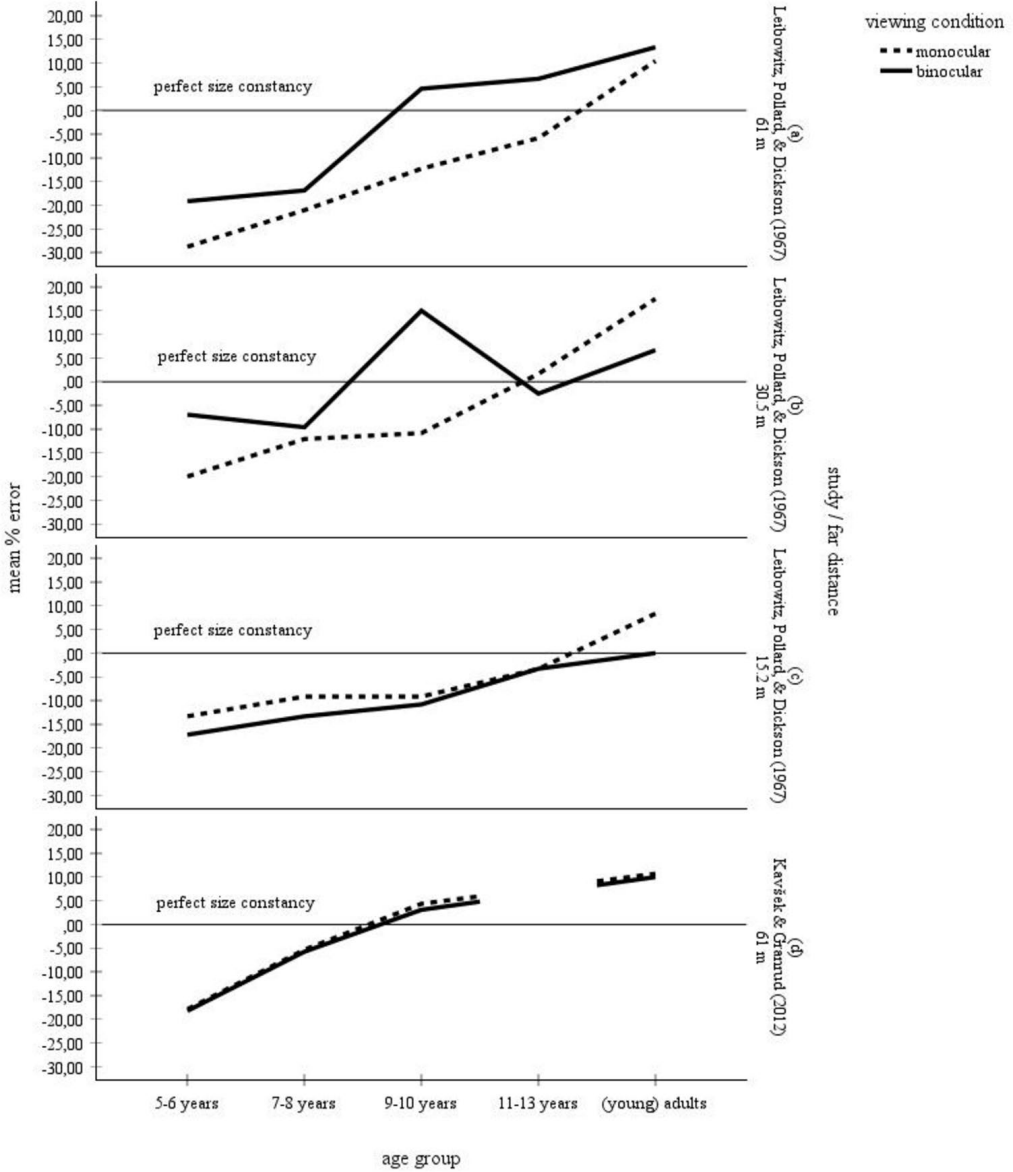
Mean error scores for the complete data set broken by monocular versus binocular viewing conditions and by age and by distance. **(a)**, **(b)**, and **(c)** show the results reported by Leibowitz et al. (1967) for the distances of 61 m, 31.5 m, and 15.2 m, respectively. **(d)** depicts the results obtained by Kavšek and Granrud (2012) for the distance of 61 m. No error bars were entered, because no corresponding information was available for the Leibowitz et al. (1967) study

The perceptual learning theory maintains that children’s size estimations of objects at far distances are diminished when binocular depth cues are not available. This hypothesis is based on the presupposition that sensitivity to monocular depth cues is immature in childhood (e.g., Yonas & Hagen, 1973). In contrast, in adults, binocular and monocular cues deliver equivalent depth information. Therefore, no monocular–binocular divergence can be established in their far distance size judgments. Figure 7 displays the mean error scores determined by each of the two studies for children’s and adults’ size judgments. Leibowitz et al. (1967) reported overall higher size estimates under binocular viewing than under monocular viewing in children for the distances of 61 m, 30.5 m, and 15.2 m. In adults, viewing condition had no effect on the size judgments. Figure 7a, b, and c depict the size estimates determined by Leibowitz et al. (1967) broken down by the 61-m, 30.5-m, and 15.2-m distances, respectively. According to Fig. 7a, the participants of all age groups made lower size estimations when viewing objects at a distance of 61 m with one eye only than when viewing the objects with both eyes. Figure 7a also indicates that this monocular–binocular difference was mainly caused by the children ages 9–10 years. At that age, binocular size estimates strongly increase and become slightly overconstant. Under monocular viewing conditions, perfect size constancy is reached by about 11–13 years of age. Figure 7b depicts the results for the far distance of 30.5 m. Like Fig. 7a, the curves in Fig. 7b confirm that the overall significant gap between binocular and monocular size perception reported by Leibowitz et al. (1967) was obviously due to the behavior of the children ages 9–10 years who displayed overconstancy under binocular viewing conditions and underconstancy under monocular viewing conditions. In the children ages 7–8 years and 11–13 years, no monocular–binocular difference occurred. At the distance of 15.2 m, according to Fig. 7c, the binocular-monocular difference was minimal for all age groups. Overall, according to the data outlined in the Fig. 7a, b, and c, the monocular–binocular divergence reported by Leibowitz et al. (1967) is largely due to a sharp increase in the binocular size estimates at the 30.5-m and 61-m distances at 9–10 years of age that is not paralleled by the monocular size estimates. The reason for this trend is unclear. All in all, these more detailed descriptions of the Leibowitz et al. (1967) data call into question the perceptual learning theory. These doubts are reinforced by the study conducted by Kavšek and Granrud (2012), who found no effect of monocular versus binocular viewing conditions on the size estimates made by children ages 5–10 years and by adults for the 61 m far distance. In fact, Fig. 7d illustrates that the size judgments under both viewing conditions were basically identical. Moreover, from 9–10 years of age onward, the size judgments tended to be overconstant.

## General discussion

### The role of age and distance

Research on the development of size constancy has a long tradition. Descriptive research has been published as early as the mid-1920s (Beyrl, 1926; Frank, 1926; see also Burzlaff, 1931) and has then been continued in the 1940 s (e.g., Lambercier, 1946a, 1946b; Piaget & Lambercier, 1943a, 1943b). A further surge in research took place from the mid-1950s to the end of the 1970 s (e.g., Cohen et al., 1958; Rapoport, 1969; Teghtsoonian & Beckwith, 1976; Tronick & Hershenson, 1979; Zeigler & Leibowitz, 1957). Nevertheless, several studies have been conducted in the subsequent period as well (e.g., Granrud & Schmechel, 2006; Shallo & Rock, 1988; Sperandio, 2021).

Interestingly, while these studies have focused on the development of size constancy in childhood, another line of research has concentrated on size constancy in infancy. Age of the participants is not the only difference between these two research areas. Another difference concerns the method of data collection. In the size-matching studies with children, the participants were asked to assess the size of a standard object by means of comparison objects. Research with infants applies looking methods—that is, the visual habituation–dishabituation method. Moreover, unlike the research with children, size constancy in infants has been assessed with objects that are very close—that is, that are positioned less than approximately 2 m from the baby.

The experimental studies with babies generally observed size constancy in infants 4 months of age and older (e.g., Day & McKenzie, 1981; Granrud, 2006; McKenzie et al., 1980). One study established size constancy even in newborns (Slater et al., 1990). Unfortunately, these studies used different object sizes and different object distances from the infant, making them difficult to compare. Moreover, in these investigations, measures were taken to ensure that the infants responded to distal, physical rather than retinal object size, that is, that the infants’ looking behavior was indeed based on size constancy. However, Granrud (2006) criticizes that the existing studies on size constancy in infancy do not allow any conclusions to be drawn about the relative weighting of physical and retinal size. In his study, Granrud (2006) tested whether infants 4 months of age respond primarily to physical or to retinal size. For example, in one experimental condition, the participants were habituated to an object with a diameter of 10 cm. The object was located at a distance of 50 cm from the participant. Retinal size or, rather, visual angle of the object was 11.31°. During the subsequent dishabituation period, the 10-cm object was presented together with an object with a diameter of 6 cm. Both objects were presented side by side, the distance to the infant was reduced to 30 cm. Such, one dishabituation object had the same physical size (10 cm), but a novel retinal size (18.44°) as the habituation object, and the other dishabituation object had a novel physical (6 cm), but the same retinal size (11.31°) as the habituation object. The infants looked longer at the dishabituation object with the novel physical size (6 cm), indicating that they recognized the 10-cm dishabituation object as familiar despite its novel retinal size and recovered their visual attention to the dishabituation object with the novel physical size (6 cm) despite its familiar retinal size. This result provides evidence that sensitivity to the constancy of physical size across different retinal sizes, that is, size constancy, is present from the first months of life onward. However, size constancy in infants was investigated with very close distances only.

The present meta-analysis dealt with size-matching studies that have assessed size constancy in children. These studies used widely varying ages, distances, and methodological variations. The goal of the meta-analysis was to discuss the effect of these varying aspects on children’s size estimates and to reveal consistent behavioral patterns within the data material of the existing studies.

Figure 2 and Tables 1 and 3 depict the distribution of size judgments across various age groups and object distances. Age of the participants clearly interacts with object distance. In younger children, an increase of distance results in a conspicuous increase of size underestimation. In older children and in adults, the effect of distance on the size estimations becomes smaller. Furthermore, size estimation accuracy clearly increases with increasing age: the underestimation of size abates, and adults are quite accurate at all distances. The age differences in size estimation accuracy are greatest at the farthest distances: at distances greater than 10 m, size estimation accuracy shows a steep improvement with age. Steepness of the developmental curve decreases with decreasing object distance.

Size estimation accuracy is highest for all age groups at distances of less than 3 m. With the exception of the children younger than 5 years of age, there are only very slight underestimation errors in childhood. Adults even slightly overestimate object size.

In sum, size constancy appears to be present at birth, but is restricted to very near distances in infancy. It then extends to distances < 3 m, where it reaches a high degree of accuracy at 5 years of age. With higher distances, retinal image size distorts size estimations during childhood.

### The proximal versus constancy mode theory

To explain children’s failure to make accurate size judgments at far distances, the proximal versus constancy mode theory assumes that children, unlike adults, rely primarily on retinal object size. In order to verify the theory, Shallo and Rock (1988) manipulated the visual angle of the comparison stimuli. According to their experimental results, younger children’s assessments of the size of the standard object that was 61 m away were underconstant when the visual angles of the comparison stimuli varied, but approached constancy when the visual angles of the comparison stimuli were all the same. The authors argue that children rely primarily on retinal stimulus information. The authors argue that when the comparison stimuli have different visual angles, children have a strong bias to conduct a retinal size matching. They therefore select a comparison object that is smaller than the distant standard object. When the comparison stimuli have equal visual angles, children are distracted from proximal stimulus information and are pushed into judging on the basis of real, physical size. However, Granrud and Schmechel (2006) could not replicate the differential effect of the arrangement of the comparison stimuli. Instead, young children underestimated the size independent of whether or not the comparison objects subtended equal visual angles. Granrud and Schmechel (2006) found no explanation for the differential results obtained by the two studies. They emphasize that the finding of underconstancy at far distances up to 61 m in children younger than 10 years has been confirmed by numerous studies. Nevertheless, further research is required to clarify the circumstances contributing to the divergent results of Shallo and Rock (1988) and Granrud and Schmechel (2006).

### The metacognitive theory

The developmental pattern in Fig. 7d found by Kavšek and Granrud (2012) is consistent with the metacognitive theory (e.g., Granrud, 2004, 2009, 2012; Rapoport, 1967) on the overall observation that young children perceive and describe distant objects as smaller as their actual size. The theory rejects the idea that size judgments are dependent on age-related improvements in responsiveness to perceptual depth cues (e.g., texture gradients, horizontal disparity, motion parallax). With increasing distance, retinal object size decreases. As young children make size estimations predominantly on the basis of the retinal image, their size matches are increasingly underconstant. After 5–6 years of age, however, children become increasingly aware of the effect of distance on perceived size. They therefore begin to correct the distance-dependent decrease of retinal size using cognitive strategies such as the distance compensation strategy. This strategy triggers an enlargement of estimated object size to compensate for the diminished perceived size. Due to the use of the distance compensation strategy, size estimates at far distances are generally accurate or even overconstant from 9–10 years of age onward. Given the summary data outlined in Table 1 and Fig. 2, however, the age from that on children make mature size constancy judgments is less clear. Overall, according to Table 1 and Fig. 2, children ages 9–10 years and even children ages 11–13 years still underestimate size at far distances and have not yet reached the adult-level. Obviously, the steady increase of the curves in Fig. 2 suggests that the improvement of size constancy with age is a continuous developmental process with a strong thrust at the age of about 5–6 years. Furthermore, the results of the studies are highly variable. For example, for the age range of 9–10 years, Brislin and Leibowitz (1970) found an underconstancy of about 20%. In contrast, Kavšek and Granrud (2012) established an overconstancy of about 3%.

The metacognitive theory has been supported and elaborated by studies conducted by Granrud (2009) and by Merriman et al. (2010). According to these studies, children begin to employ the distance compensation strategy as early as 5–6 years of age. Consistent with both studies, the meta-analytical data established a strong increase in the accuracy of far distance size estimations at about 5–6 years of age.

In sum, the metacognitive theory is consistent with the metanalytical observation of a substantial improvement of size constancy after about 5 years of age. Granrud (2009) stressed that by the age of 9–10 years, most children deliberately use the distance compensation strategy. Their size constancy ability is therefore quite mature. Table 1 and Fig. 2, however, imply that there are still improvements even beyond the age of 11–13 years.

### The perceptual learning theory

As an explanation of younger children’s tendency to underestimate size at far distances, the perceptual learning theory (e.g., Jenkin & Feallock, 1960; Leibowitz, 1974; Leibowitz et al., 1967) suggests that pictorial cues to depth are crucial for size perception and that children are less sensitive to these cues. In support of the perceptual learning theory, Leibowitz et al. (1967) experimentally established a difference between monocular and binocular size estimations at far distances. More specifically, according to Leibowitz et al. (1967), participants ages 5–9 years made smaller size matches at far distances when viewing the stimuli with one eye only than when viewing them with both eyes. In contrast, adults made nearly accurate size estimates under both monocular and binocular viewing conditions. According to Leibowitz et al. (1967), size constancy is fully developed in adults, unlike in children, and they are able to make full use of both binocular and monocular cues to depth. As a result, there is no difference between monocular versus binocular viewing conditions in adults. Indeed, several studies found that responsiveness to pictorial depth cues develops over childhood (e.g., Jahoda & McGurk, 1974; Olson & Boswell, 1976; Wilcox & Teghtsoonian, 1971; Wohlwill, 1962; Yonas & Hagen, 1973). On the other hand, sensitivity to pictorial cues to depth is present even in infants (e.g., Kavšek et al., 2009; Oross et al., 1987). Moreover, the findings with children are somewhat inconsistent. In general, research has observed an age-related improvement of the ability to exploit pictorial information for distance and size. However, some of the reported age effects are quite small. For example, Wohlwill (1962) describes that sensitivity to perspective cues is largely adult-like in children ages 6–7, 9–10, and 13–14 years. Jahoda and McGurk (1974) found an improvement in responsiveness to relative height, linear perspective, and texture gradients between 4 and 10 years of age. However, even the youngest children’s ability to estimate object size using pictorial cues was relatively high. Most importantly, contrary to the results of Leibowitz et al. (1967), Kavšek and Granrud (2012) observed no effect of monocular versus binocular viewing conditions on the far distance (61 m) size estimations in children 5–10 years of age and in adults. This result, which is shown in Fig. 7d, clearly contradicts the perceptual learning theory. Figure 7a, b, and c portray the data obtained by Leibowitz et al. (1967) for different far distances (15.2 m, 31.5 m, and 61 m, respectively). The figures illustrate that children’s monocular far distance size estimations are not inferior to their binocular estimations in general. It should be noted, however, that the presentation of the Leibowitz et al. (1967) data in Fig. 7 is subject to the proviso that the entered scores are estimated from graphs that only provide imprecise scaling. Future research has to further clarify the impact of monocular depth cues. Also, the role of binocular depth information at close distances (< 6 m) should be examined in detail. Especially, it should be tested whether binocular viewing provides an advantage at nearer distances, because disparity and convergence of the eyes are most effective at near distances (e.g., Cutting & Vishton, 1995).

Since the discussion of the perceptual learning theory includes the factor of monocular versus binocular viewing conditions, this experimental factor is not analyzed further below.

### Mode of presentation of the comparison objects: Single versus series presentation

The results of the meta-analysis suggest that some methodological variations affect estimated size while others do not. Underconstancy in children is more pronounced when the entire series of comparison objects is presented singly—that is, one after another than when the comparison objects are presented together (see Table 3 and Fig. 3). Especially, when using the single presentation mode, at distances > 3 m, underconstancy in children clearly increases with increasing distance. With the series presentation mode, this effect is far less pronounced. Presumably, the possibility to not only compare the standard with each individual comparison object one after the other but to compare all comparison stimuli simultaneously with the standard and also amongst each other exerts a facilitating effect on children’s size judgments. For instance, Shallo and Rock (1988) compared the effect of presenting the comparison stimuli singly versus all together. The study found an improved size constancy with the series than with the single presentation method in children ages 4–6 years at a distance of 61 m. In adults, the size judgments were slightly underconstant with the single presentation method and slightly overconstant with the series presentation method. In general, according to Fig. 3, adults’ judgments are quite accurate with both methods. For distances > 10 m, the single presentation method results in a slight underconstancy, whereas the series presentation mode engenders a slight overconstancy (e.g., Burzlaff, 1931; Lambercier, 1946a; Shallo & Rock, 1988).

Burzlaff (1931) tested children ages 4–7 years for their size constancy in a near distance of 4 m. He found a slight size underestimation using the single presentation method and a very slight overconstancy using the series presentation method. Furthermore, there were only small age differences. Lambercier (1946a) conjectures that the higher size constancy for the series presentation method in Burzlaff’s (1931) study might be a central tendency effect. More specifically, a series presentation of the comparison stimuli in order of size, as in Burzlaff’s(1931) study, might produce a preference for the middle of the object series. As the middle comparison object in that study had approximately the same size as the standard stimulus, the series presentation mode entailed less underconstancy and more constant size judgments in children than did the single presentation method. To support this argumentation, Lambercier (1946a) experimentally presented different series of comparison objects with different midpoints. Indeed, his results confirmed a central tendency effect. Wohlwill (1963), however, raised objections to these findings by pointing out a potential bias in Lambercier’s (1946a) data. In some of the series of comparison objects in the Lambercier (1946a) study, the comparison object that physically corresponded to the size of the standard was very close to the comparison object with the largest size. Due to this special arrangement, several participants were excluded from data analysis, because they found no matching comparison and therefore delivered no data. As a result, the data were biased towards the middle part of the comparison series. This criticism points to the need for careful selection of the stimulus material. Nevertheless, it does not affect the usefulness of the method of a joint presentation of the comparison items in general. For example, Kavšek and Granrud (2012) presented standard objects of different sizes in a near (6.1 m) and in a far (61 m) distance. Children and adults were asked to compare these standards with nine comparison objects arranged in order of size 2 m away from the participant. The smallest object was on the left and the largest was on the right from the participant’s point of view. The selected matching comparison objects clearly reflected the variation in size within the standard objects. Moreover, the selected comparison objects were distributed over the right half of the arrangement of the comparison objects. There was no discernible central tendency effect.

### Angular separation between standard and comparison objects: Simultaneous versus nonsimultaneous visibility of the standard and the comparison stimuli

Another methodological manipulation concerns the angular separation between standard and comparison. The angular separation between the experimental stimuli can be so large that the standard and comparison objects can no longer be detected at a glance—that is, that the participant has to look back and forth in order to compare them. Alternatively, the angular separation can be made so small that all objects are simultaneously visible. For small distances, Piaget and Lambercier (1943a) observed a slight underconstancy for a small stimulus separation and a slight overconstancy for a large stimulus separation in children. Adults displayed an overconstancy for both angular separations. Moreover, overconstancy was higher for the small angular object separation.

The descriptive analysis of the meta-analytical data set depicted in Table 4 and in Fig. 4 suggests that children display underconstancy with both large and small angular separations between comparison and standard objects. Moreover, in the children ages 9–13 years, distance exerts a smaller effect on the size estimations under large angular stimulus separations than when all experimental objects can be viewed at a glance. The statistical analysis of the data in Table 4 established no significant main effect of the angular separation variable. These observations contradict Brislin’s and Leibowitz’s (1970) finding that small angular stimulus separations foster size constancy.

**Fig. 8:**
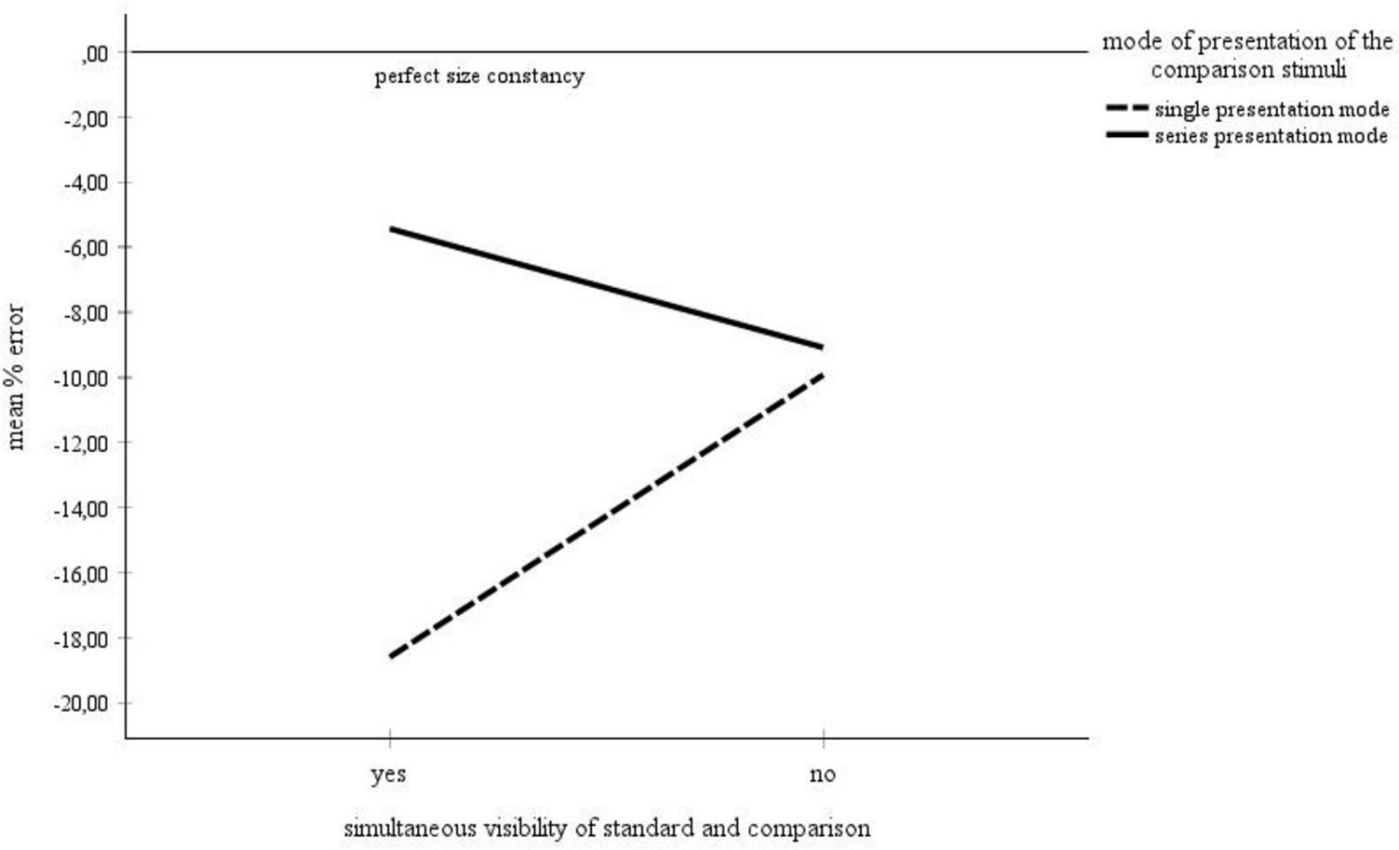
Interaction between mode of presentation of the comparison stimuli (single versus series presentation of the comparison objects) and angular separation between comparison and standard stimuli (simultaneous versus nonsimultaneous visibility of standard and comparison). The data are collapsed across all age groups and all distances > 5 m. No error bars were entered, because no corresponding information was available

According to the statements made so far, children show less underconstancy at greater distances when all comparison objects are presented together. On the other hand, children’s size judgments show clearer distance differences when the standard and comparison objects can be viewed at a glance. Interestingly, as illustrated in Fig. 8, both variables strongly interact. Figure 8 contains the mean size errors for distances greater than 5 m of all age groups. If standard and comparison can be captured at a glance, an additional joint presentation of all comparison objects triggers a smaller size underconstancy or, rather, a better size constancy (− 5.43%) than a successive presentation of the comparison items (− 18.60%). In contrast, if standard and comparison cannot be viewed simultaneously, it does not matter whether or not the comparison objects are presented singly or together. In both cases, underestimation amounts to about − 9.5%. The possibility to monitor all experimental objects at once hence drives the size judgments towards constancy. However, if there is always only one comparison object together with the standard object in the common field of view, size judgments are strongly biased towards retinal size, which leads to a strong underconstancy. If the participant has to look back and forth to compare standard and comparison, mode of presentation of the comparison objects is irrelevant and size judgments are of medium underconstancy.

### Relative position of standard and comparison objects: Comparison objects nearer versus farther away than standard objects

In his overview, Wohlwill (1963) points out that the relative position of standard and the comparison objects affects adults’ size estimations. More specifically, when the comparison objects are close to the participant, while the standard object is further away from the participant, adults exhibit consistent overconstancy. Reversion of position of standard and comparison produces a lower overconstancy or even a slight underconstancy (e.g., Akishige, 1936; Piaget & Lambercier, 1943a). Table 5 and Fig. 5 confirm this observation, although the trend is rather weak. Children ages 5–8 years make less underconstant errors when the comparison objects are closer to them than the standard object. In the case of farther away comparison objects, the nearby standard has to be compared with objects that produce different retinal sizes. The determination of size constancy therefore involves an adjustment of several retinal images. Presumably, this is more challenging than the reverse situation with nearby comparison objects and a distant standard object, where only one retinal image needs to be assessed. As a consequence, children ages 5–8 years exhibit a stronger underconstancy in the first situation than in the latter one.

### Kind of experimental size instructions: Objective versus apparent size instructions

The impact of objective versus apparent size instructions has been focused by Granrud (2009), Piaget and Lambercier (1951, 1956) and Rapoport (1967). According to these studies, instructions exert a significant effect. In general, participants of all ages underestimate size to a higher degree when instructed to judge the apparent, perceived size than when asked to judge the objective, real size (see Table 6 and Fig. 6). This finding is consistent with earlier studies with adults only (e.g., Carlson, 1960; Leibowitz & Harvey, 1969). Children ages 7–8 years and older and adults have very low negative mean error scores in the 3–5-m distance category under apparent size instructions. This extremely pronounced underconstancy arises from the data material in the Piaget and Lambercier (1951, 1956) studies.

Interestingly, Granrud (2009) found that only children who were aware of the relation between distance and retinal size understood the distinction between objective and apparent size. When asked to select the comparison object that matched the objective, real size of a distant (61 m) object, they inflated their size estimations and were able to estimate the accurate size of the object by applying the size compensation strategy. When asked to indicate the apparent size of the standard object—that is, the size the standard object appeared to be, they underestimated size. In contrast, children who had no knowledge about the connection between distance and apparent size and about the size compensation strategy, underestimated size with both objective and apparent size instructions. At the near distance of 6.1 m, children underestimated size under both objective and apparent size instructions independent of whether or not they had knowledge about the effect of distance on perceived, retinal size. Obviously, at near distances, children generally believe that apparent size is correct. These observations suggest that even if children have knowledge of the distinction between apparent and objective size, they do not apply this knowledge at a near distance (see also Merriman et al., 2010).

### Concluding considerations

Table 1 and Fig. 1 illustrate that before the age of 5 years, children’s near and far size estimations are clearly underconstant. Perhaps, the sharp increase in size constancy that occurs before and up to the age of 5–6 years for all distances reflects the effect of a developmental increase in sensitivity to pictorial and binocular depth signals (see also Kubzansky et al., 1971). Indeed, several studies provide evidence for an improvement of depth perception beyond infancy. Processing of binocular depth, which is especially important for near distance perception, improves until 5–7 years of age and beyond (e.g., Norcia & Gerhard, 2015, 2017; Simons, 1981). Also, pictorial depth perception improves during childhood (e.g., Jahoda & McGurk, 1974; Yonas & Hagen, 1973). From the age of 5–6 years onward, size constancy development might be additionally supported by emerging cognitive compensation strategies. This assumption combines the perceptual learning theory (e.g., Leibowitz et al., 1967) with the metacognitive theory (e.g., Granrud, 2004, 2012). Cognitive correction strategies play a decisive role in estimating the size of distant objects. At very close distances (< 3 m), cognitive factors might play a subordinate role during development. Here, size judgments might reflect an implicit, automatic perceptual computation of size from the combination of proximal and distance information. This implicit automatic size estimation process might be also functional in infancy. Indeed, size constancy has been found for distances < 2 m in infants (e.g., Granrud, 2006). The crucial depth cues employed by young infants are assumed to be convergence and accommodation of the eyes (e.g., Kellman, 1995). Convergence and accommodation have been shown to contribute significantly to size constancy at very short distances in adults (e.g., Leibowitz et al., 1972b). They mature rapidly during the first months of life (e.g., Horwood & Riddell, 2013). During the course of infancy, convergence and accommodation are supplemented by the emerging abilities to exploit kinetic, binocular, and pictorial cues to depth (for a summary, see Arterberry & Kellman, 2016). From 5–6 years of age onward, mean size errors for distances < 3 m meander slightly below perfect size constancy. Presumably, at 5–6 years of age, processing of depth cues is mature enough to enable nearly perfect size constancy at distances < 3 m.

Research on size constancy in adults only has not been included in this developmental overview. The relevant research found overconstancy in adult samples (see Wohlwill, 1963). According to Table 1, however, size constancy in adults is quite perfect. Table 5 and Fig. 5 indicate that the observation of overconstancy might be an effect of the relative position of the experimental stimuli. The mean error scores are generally higher, when the comparison objects are nearer than the standard than when the standard is nearer than the comparison (e.g., Jenkin & Feallock, 1960; Piaget & Lambercier, 1943a). In adults, this effect results in overconstant judgments for all distances in case the comparison is closer than the standard.

The findings on the development of size constancy during adulthood are sparse. For the near distance of 6.1 m, Kavšek and Granrud (2012) found an underconstancy of − 2.47% in adults ages 19–28 years of age and of − 6.06% in adults older than 50 years of age. For the far distance of 61 m, size estimation errors for the two age groups amounted to 10% and 3.61%, respectively. Although these scores indicate a somewhat stronger underconstancy for the near distance and a somewhat less pronounced overconstancy for the far distance in older adults, the differences between age groups were not significant. One task of future research is to more thoroughly investigate the developmental course of size constancy during adulthood.

This meta-analysis concentrated on size-matching studies. An alternative method has been proposed by Sperandio (2021; see also Sperandio et al., 2009). Sperandio et al. (2009) established that detection time is modulated by perceived object size. Using this observation, Sperandio (2021) tested the size constancy performance in children ages 5–14 years and in adults. According to the results, for all age groups, reaction times decreased with increasing distal object size but were not affected by retinal object size. Sperandio (2021) concluded that size constancy is present in children 5 years of age. Maximal object distance was set to 114 cm. The study thus extends the observation of size constancy in infancy for very near viewing distances to children. One challenge for future research on size constancy will be the inclusion of children in the age range between infancy and preschool age. Moreover, distances less than 3 m should be investigated more systematically.

In sum, the results of size-matching studies are strongly influenced by the experimental design such as a single versus series presentation of the comparison objects. Nevertheless, the overall overview of the data results in the developmental trajectory depicted in Fig. 2. Three theories compete to explain the development of size constancy in childhood. The perceptual learning theory stresses the impact of the emerging sensitivity to depth cues (Leibowitz et al., 1967). The proximal versus constancy mode theory claims that children’s size estimates are strongly biased by a tendency to respond to retinal size (Shallo & Rock, 1988). The metacognitive theory highlights the impact of explicit cognitive strategies that inflate the diminished retinal size of objects at far distances. These theories thus focus different aspects of the visual system’s ability to combine retinal image and distance cues in such a way that objects are attributed a coherent size regardless of their distance. A mere comprehensive theory should include all aspects mentioned in the theories, and be able to explain the effects of methodological variations. It should also take research on the physiology of size constancy into account. This research provides evidence that cortical structures such as the visual areas V1 and V4 (e.g., Chen et al., 2019a, 2019b; Sperandio & Chouinard, 2015; Tanaka & Fujita, 2015) are involved in the processing of size and distance or, rather, in the encoding of actual object size. Future research should also collect and compare neurophysiological measurements from different age groups.

## Data Availability

The dataset generated and analyzed during the current study is available in the OSF repository: https://osf.io/4r69f/files/sufw4?view_only=16f6ac8055fc4a10b316214e0742adb6 The review and meta-analysis was not preregistered.

## Declarations

### Open practices statement

The review and meta-analysis was not preregistered.
